# Force Measurements From Myofibril to Filament

**DOI:** 10.3389/fphys.2021.817036

**Published:** 2022-01-27

**Authors:** Steven Marston

**Affiliations:** NHLI, Imperial College London, London, United Kingdom

**Keywords:** contractility, myofibril contractility, thick filament, thin filament, tension measurement

## Abstract

Contractility, the generation of force and movement by molecular motors, is the hallmark of all muscles, including striated muscle. Contractility can be studied at every level of organization from a whole animal to single molecules. Measurements at sub-cellular level are particularly useful since, in the absence of the excitation-contraction coupling system, the properties of the contractile proteins can be directly investigated; revealing mechanistic details not accessible in intact muscle. Moreover, the conditions can be manipulated with ease, for instance changes in activator Ca^2+^, small molecule effector concentration or phosphorylation levels and introducing mutations. Subcellular methods can be successfully applied to frozen materials and generally require the smallest amount of tissue, thus greatly increasing the range of possible experiments compared with the study of intact muscle and cells. Whilst measurement of movement at the subcellular level is relatively simple, measurement of force is more challenging. This mini review will describe current methods for measuring force production at the subcellular level including single myofibril and single myofilament techniques.

## Introduction

The production of force and movement by molecular motors is the essence of contractility. To understand the basic mechanisms of contraction and Ca^2+^ regulation we need to isolate the contractile elements from the physiological Excitation-Contraction (EC) system and from other, mainly parallel, mechanical elements that are present in whole muscle.

Contractility is usually measured in intact small muscle strips or single myocytes where contractility is dependent on the EC coupling system and neural and humoral stimulation that act on the Ca^2+^-regulation of the contractile apparatus itself. Chemically skinned muscle offers a way to study Ca^2+^ regulation of contractility in isolation from EC coupling but retains confounding effects of parallel elasticity. Moreover, the usefulness of skinned fiber studies is limited by slow diffusion of solutes into the fiber that rules out dynamic measurements. We therefore need to study contractility at the subcellular level.

Contractility measurements at the sub cellular level in myofibrils and single filaments offer advantages that make their development worthwhile. Solution conditions are easy to manipulate and rapid (i.e., 10 ms) solution changing is possible since the solution diffuses rapidly into the myofibril so that the kinetics of the contractile response to rapid concentration jumps may be measured. There is no limitation on the source material that can be used and the quantities required are very small; myofibrils and myofilaments can be isolated from small samples (i.e., 20 mg) of muscle or cells. Fresh or frozen tissue are equally good sources and post-translational modifications can be changed or proteins substituted prior to measurement. Finally, since all measurements are made under the microscope it is possible to combine force measurements with additional video microscopy techniques such as observing sarcomeric structure dynamics and the turnover of fluorescent substrates.

To extend force measurements from muscle to single myofibrils or even single filaments requires a number of technological advances that were exceptional in the 1990s when these methods were first developed but have now become mainstream. Nevertheless, handling tiny samples is still challenging and is far from routine: whilst it is now easy to measure small forces, the problem of how to attach myofibrils and filaments to the measuring device is the greatest obstacle to high throughput experimentation.

In this review I will describe the current methodologies for single myofibril and single filament force measurements. At the time of writing none of the techniques described here can be bought as turnkey systems; to do such studies it is necessary to be able to build your own apparatus.

The principal of all force measurements is that the contractile element is attached to probes at both ends such that length changes can be imposed and force may be measured. The critical requirements for such measurements is that the contractile element is firmly attached to the probes and that the tension sensing probe be sufficiently compliant to enable probe movement to be translated into a signal yet sufficiently rigid that the contractile element is essentially isometric. The methods developed usually involve glass microneedles, AFM cantilevers or a stiff optical trap as the sensor. Table 1 summarizes the various methods that have been published.

**TABLE 1:** Techniques for single myofibril and single filament force measurements.

References	Attachment method	Force transducer
**Myofibrils**		
Bartoo et al., 1993	Silicone adhesive (Dow 3145 RTV)	Glass microneedle with split diode detector
Colomo et al., 1997	Myofibrils adhered directly to glass	Glass microneedle with split diode detector
Stehle et al., 2002	Silicon adhesive (Dow 3140 RTV)	Force modulation etched silicon probe-type atomic force cantilever (Nanosensors). Detected by the displacement of reflected beam
Pavlov et al., 2009	Glass microneedles inserted into myofibril	Microneedle movement detected by linear photodiode array (10,680 pixels)
**Single filaments**		
VanBuren et al., 1994a,b	Glass microneedle coated with HMM	Microneedle deflection detected by video microscopy
Holohan and Marston, 2005	Magnetic beads coated with gelsolin	Electromagnet and video microscopy
Kaya et al., 2017	Myosin/rod copolymer on glass surface, actin-biotin phalloidin TRITC attached to streptavidin coated bead	Optical trap detected by feedback needed to keep bead steady under load
Pertici et al., 2018	HMM on glass microfiber coated with nitrocellulose, actin attached to gelsolin coated bead	Optical trap detected by feedback needed to keep bead steady under load
Bing et al., 2000	a-actinin attached to cover glass	Indirect: quantity of a-actinin that stops actin filament movement
Cheng et al., 2020	Cantilever coated with a-actinin to specifically attach actin filament. Myosin adheres spontaneously	Microfabricated silicon nitride cantilever bending

## Single Myofibrils

Measurement of force in single myofibrils has become the main technique used to study contractility at the subcellular level. Iwazumi (1987) published the earliest studies on myofibril contraction based on an electromagnetic force transducer and this methodology was used by Friedman and Goldman (1996) with some success but low throughput. A more versatile technique for measuring force in single myofibrils, based on glass microneedles, was first described by Bartoo et al. (1993) and was developed by Colomo et al. (1997) for active force measurement and by Linke et al. (1997) for passive force measurement. The principle of using glass microneedles for force measurement was introduced by Kishino and Yanagida (1988). More recent accounts (Ayittey et al., 2009; Vikhorev et al., 2015) describe how to make and calibrate the bent microneedles capable of measuring force in the 0.1–0.3 μN range typical of single myofibrils. The prepared cantilever force probes have a compliance of 2–17 mm/mN when deflected by small forces pulling perpendicularly.

**FIGURE 1:**
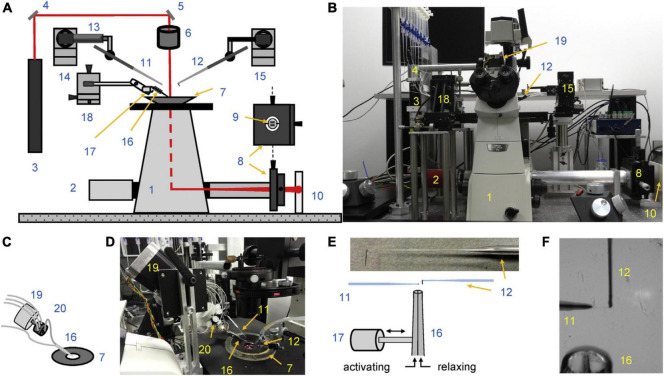
Experimental setup. **(A,B)** Schematic overview **(A)** and photograph **(B)** show the key elements of the apparatus. **(C,D)** Schematics **(C)** and photograph **(D)** of multi-solution change system. **(E)** Schematics of cantilever and ultrafast solution change system. The left-sided microtool is relatively stiff and is mounted onto a piezo actuator for fast myofibril length change. The right-sided glass microtools is a high compliance L-shaped cantilever that acts as a force sensor. The inset shows a photograph showing the shape of the cantilever tip. **(F)** The photograph shows two microtools holding a small myofibril bundle. The relaxing and activating solutions are applied via the adjacent barrels of a double-barreled (Q) micropipette. The numbers on the figure indicate: 1, inverted microscope; 2, camera; 3, laser; 4 and 5, mirrors; 6, microscope condenser; 7, bath mounted onto a microscope stage; 8, XY positioning stage; 9, segmented photodiode; 10, projection plane for visual control of cantilever positioning; 11, left microtool; 12, right microtool (cantilever force sensor); 13, piezo actuator; 14 and 15, left and right micromanipulators for the positioning of left and right microtool, respectively; 16, double-barreled Q pipette; 17, stepper motor controlling the position of the double-barreled Q pipette; 18, XYZ positioning stage for the ultrafast two solutions switching system; 19, stepper motor controlling the valve opening of the eight-channel valve; 20, eight-channel valve. Reproduced from Vikhorev et al. (2015) with permission.

This methodology that has been adopted by a number of laboratories. An alternative measurement technique based on an AFM cantilever force detector produces broadly similar results (Stehle et al., 2002). The microneedle-based methodology was thoroughly documented by Vikhorev et al. (2015) see Figure 1. The heart of the instrument is the two microneedles. Myofibrils attach direct to glass and just touching the needle to the end of a myofibril results in firm adherence. In order to maximize the surface of myofibril attached to the probes they are manipulated to increase the myofibril wrapping around the microneedles. The tension transducer microneedle is crucial; the probe needs to be made in such a way that it is compliant enough to give sufficient bending under tension to be accurately measured in a position detector such as a split photodiode detector, yet stiff enough to keep the contracting muscle near isometric.

These force measurement methods are both sensitive and accurate, with high time resolution and low noise. They can be combined with rapid manipulation of length and uniquely can be used with fast solution changing and video microscopy of the sarcomere pattern, thus enabling a wide range of protocols that are not possible with large muscle preparations.

**FIGURE 2:**
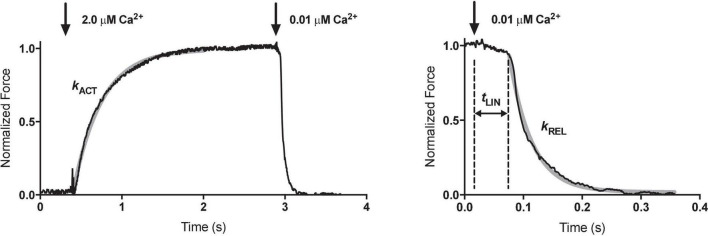
Measurement of contractility in single mouse cardiac muscle myofibrils. Time course of contraction and relaxation. The arrows indicate the time when the solutions were switched. The kinetic parameters maximum force (F_max_), rate of force development (k_ACT_), duration (t_LIN_) and slope (k_LIN_) of the slow relaxation phase, and rate of the fast relaxation phase (k_REL_) were determined from the traces. The slow-relaxation phase was counted from the initiation of micropipette movement. The beginning of the exponential phase of relaxation was considered as the end of slow-relaxation phase. The relaxation phase is shown over an expanded timescale to show the initial linear, nearly isometric force decay period tLIN, followed by an exponential relaxation with a rate constant kREL. The gray solid lines represent the best exponential fits to the data. The temperature was 17°C. Reproduced from Vikhorev et al. (2014) with permission.

**FIGURE 3:**
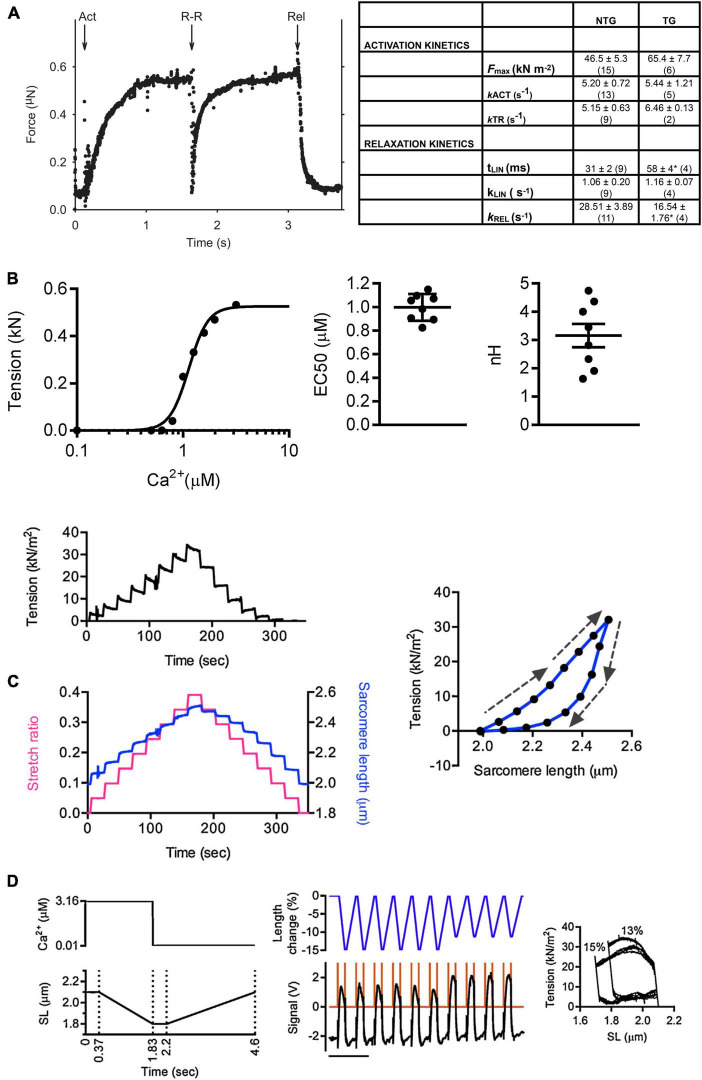
Examples of experiments with single myofibrils. **(A)** Determination of kinetic parameters in wild-type and transgenic (ACTC E99K) mouse myofibrils **(B)** Measurement of the Ca^2 +^-sensitivity of tension in mouse myofibrils. **(C)** Measurement of passive tension. **(D)** Simulated work lops. Arrows indicate stretch or release. Reproduced from Vikhorev et al. (2015) with permission.

Rapid solution changing is the most important advantage of force measurements in myofibrils. Colomo et al. (1998) introduced the moving double-barreled pipette method that is almost universally used (Figures 1E,F). The time for solution switch to complete is less than 10 ms. Since the smallness of myofibrils means that there is no diffusional lag, the rates of crossbridge attachment on a Ca^2+^ jump can be measured and the time course of relaxation when Ca^2+^ is removed can be followed (Figure 2). The absence of diffusional lag can be demonstrated by measuring the rate of crossbridge attachment following a rapid release and re-stretch – K_tr_ (Tesi et al., 2002) which is found to equal the rate of tension increase on Ca^2+^ jump (Figure 3A). Rapid removal of Ca^2+^ in myofibrils has revealed a two phase relaxation process that could not be detected in skinned fibers even with caged EGTA (Johns et al., 1999). Multiple solution changes enable dose-response curves to be generated, for instance the Ca^2+^ activation curve (Figure 3B).

The basic measurement of myofibrillar contractility is the Ca^2+^ activation-relaxation cycle (Figure 2). Maximum isometric contraction is around 100 N/m^2^. The contraction and relaxation transients represent fundamental processes of the contractile apparatus not measurable in larger preparations.

k_ACT_ represents the cross-bridge turnover rate which is determined by the dynamic equilibrium between the apparent rates with which cross bridges enter and leave the force generating states. It is dependent on the rate at which thin filaments are switched on by Ca^2+^ and is thus Ca^2+^ dependent (Vikhorev et al., 2014). Relaxation following a rapid drop in (Ca^2+^) occurs in two phases. This pattern of results is only seen in myofibrils due to the rapid diffusion of Ca^2+^ away from the very small contractile element. Relaxation is initiated by Ca^2+^ dissociating from troponin C coupled to the release of the C-terminus of troponin I from troponin C and its attachment to actin, where it blocks cross-bridge binding cooperatively. The initial slow decrease in the force trace, defined by k_LIN_ and t_LIN,_ has been ascribed to the time taken for the occupancy of cycling cross-bridges to drop below the threshold for cooperative activation of the thin filament, and the subsequent rapid-relaxation phase corresponds to the detachment of the remaining cross-bridges. t_LIN_ is shortened and k_LIN_ increased by high phosphate concentrations but lengthened by MgADP indicating a close relationship of this process with the product release step of the crossbridge cycle (Tesi et al., 2002). The interpretation of the relaxation curve and its relationship to heterogeneous sarcomere lengthening was fully described by Stehle et al. (2009). Both k_LIN_/t_LIN_ and k_REL_ are altered by physiological and pathological perturbations that affect thin filament relaxation rate such as cardiac TnI phosphorylation or HCM related mutations (Song et al., 2013; Vikhorev et al., 2014).

When fully implemented, the myofibril contractility method allows a wide range of measurement possibilities. Mechanical kinetics similar to whole muscle fiber measurements are possible with myofibrils as demonstrated by the quick release/restretch protocol used to measure k_tr_ (Figure 3A). It is feasible to reproduce most of the mechanical perturbation protocols that have been used in chemically skinned fibers, but in practice these have not been extensively explored since they do not differ greatly whilst the opportunities for transient biochemical perturbation that is possible in myofibril studies yields exciting new insights. Passive stiffness can also be measured and provides results that are wholly due to sarcomeric elements, mainly titin (Linke et al., 1997; Makarenko et al., 2004; Opitz et al., 2004; Figure 3C). Solution changing can be adapted to measure response to a range of concentrations and generate dose-response curves (Figure 3B). A number of unique complex protocols are also accessible, for instance the simulation of the heart work cycle by combining carefully timed Ca^2+^ jumps up and down with length ramps simulating “systolic” shortening and “diastolic” lengthening between isometric phases (Figure 3D). The addition of video microscopy allows sarcomere length to be measured, enabling accurate sarcomere length adjustments, for instance when measuring length-dependent activation. During relaxation these observations have shown transient heterogeneous shortening of sarcomeres in a myofibril which has important implications for the structural changes behind the relaxation process (Stehle et al., 2002, 2009).

## Applications

Because the myofibril measurements can be made with any suitable source of muscle the methodology has been applied to many systems. The dynamics of Ca^2+^ activation of force, defined by F_max_, k_ACT_, t_LIN_, k_LIN_, and k_REL_ and Ca^2+^ activation curves have been measured in myofibrils from both skeletal and cardiac muscles. Studies include:

(1) Comparing wild type with mutant muscles from transgenic mice such as the ACTC mutations E99K (HCM) and E361G (DCM) (Song et al., 2013; Vikhorev et al., 2014) or in mutant human heart myofibrils such as MYH7 R403Q, TNNT2 K280N, and titin truncating mutations (Ferrantini et al., 2009; Piroddi et al., 2019; Vikhorev et al., 2020).

(2) Based on techniques developed in skinned fibers (Brenner et al., 1999), troponin or tropomyosin can be exchanged with recombinant troponin components such as the HCM mutations TNNI3 R145G and TNNT2 K280N (Kruger et al., 2005; Piroddi et al., 2019) or tropomyosin isoforms or mutations (Siththanandan et al., 2009; Janco et al., 2012; Nixon et al., 2013; Scellini et al., 2014). Such studies have clearly demonstrated the effects of mutations on Ca^2+^-sensitivity and on the kinetic parameters.

(3) The same methodology can be applied to the effects of contractile protein phosphorylation, notably by PKA, on the Ca^2+^ sensitivity and relaxation kinetics (Vikhorev et al., 2014, 2020) and to determine the effects of myofilament targeting drugs such as Mavacamten and Omecamtiv Mecarbil (Scellini et al., 2020, 2021).

(4) The myofibril force assay is particularly useful when used with myofibrils from non-muscle sources. For instance in the study of embryonic heart, biopsies, cultured myocytes and particularly iPSC (Pioner et al., 2015, 2016; Racca et al., 2015; Iorga et al., 2018).

(5) A novel application is in measuring the effects of muscle stretch on passive elasticity and on active force generation. The former allows for assessment of titin mechanical properties (Linke et al., 1997; Makarenko et al., 2004; Opitz et al., 2004; Vikhorev et al., 2017) whilst the latter allows measurement of length dependent activation. Such studies have confirmed that both PKA-dependent phosphorylation and titin mutations can modulate length dependent activation whist having little or no effect on baseline contractility (Vikhorev et al., 2020).

(6) The single myofibril force assay has been adapted to measure force from a single sarcomere or even half-sarcomeres. Contraction-relaxation cycles, a force-velocity relationship and even Length dependent activation was successfully measured in single sarcomeres (Pavlov et al., 2009; Minozzo et al., 2013; Lowey et al., 2018).

## Single Filament Force Measurements

Measurement of contractility at the single filament level enables us to determine the characteristics of the actomyosin contractile machine in the absence of the effects of structure of the sarcomere. To go to singe filaments requires different, more sensitive techniques, generally derived from *in vitro* motility assay or optical trap methodology (see Table 1).

The *in vitro* motility assay was devised about 30 years ago (Kron et al., 1991) and has been immensely successful for assaying the unloaded contractility of muscle filaments, characterizing myosins and thin filament regulatory proteins and their post-translational modifications (Fraser and Marston, 1995; Gordon et al., 1997; Marston, 2003; Batters et al., 2014).

Adaptation of the IVMA to measure force requires a probe, usually attached to the thin filament, acting on immobilized myosin. In the first studies this was simply a bed of HMM molecules as used in normal IVMA but more sophisticated setups using whole thick filaments and introducing filament orientation have been devised.

The first studies used microneedles attached to thin filaments using NEM-HMM for attachment, developed by Kishino and Yanagida (1988) and Chaen et al. (1989). These were far more compliant than used for myofibrils since the forces obtained are in the region of 50 pN. Video microscopy is used to watch the needle bend until it stalls. The angle then represents the isometric force which is generally expressed as pN/μm length of the actin filament overlap; values in the region of 10 pN/μm actin are obtained. VanBuren successfully used this technique to determine the effects of light chain removal and of myosin and tropomyosin isoform switches on force generation (VanBuren et al., 1994a,b, 1999). Homsher et al. (2000) used this technique to demonstrate Ca^2+^ control of force in single regulated thin filaments.

**FIGURE 4:**
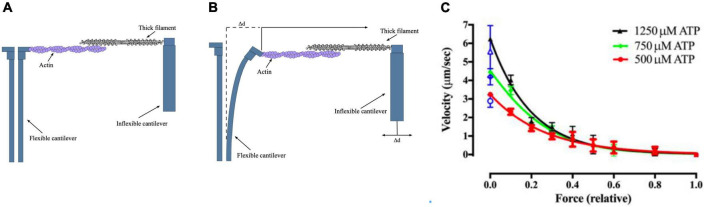
The microfabricated cantilever single filament force assay. **(A,B)** Schematic view of the microfabricated cantilever experimental setup. Thick filament and actin filaments were attached to the cantilevers and brought into contact. The filaments y produce a force that causes the displacement of the flexible cantilever, which enabled force to be measured. **(C)** Example of a single filament force -velocity plot. Figures provided by Drs Y-S Sheng and DE Rassier.

A more recent version of this methodology uses microfabricated AFM cantilevers as the tension probe (Figure 4). The stiffness of the cantilever is 0.18 pN/nm. This study also used whole thick filaments in the correct orientation for optimum interaction with thin filaments. These measurements obtained values between 60 and 100 pN/μm which may more closely indicate the real maximum force generated by myosin motors. Since the inflexible cantilever can be moved to compensate for muscle shortening it is possible to make further measurements, such as a force-velocity plot, which closely resembles that of intact muscle (Cheng et al., 2020).

Another method used for imposing load on a single actin filament is by magnetic force. The actin filaments are attached to magnetic beads via gelsolin in a normal IVMA cell and an electromagnet applies a magnetic force. Bead-tailed filaments moving over a bed of HMM slowed down under an increasing magnetic load, eventually stalled and then slid backward under increasing load before detaching from the surface. Single-filament force–velocity curves were constructed and a stalling force of about 600 pN/μm of actin filament estimated. This method has not seen much use due to the very low yield of viable bead-tailed filaments (Bershitsky et al., 1996; Hamelink, 1999; Holohan and Marston, 2005).

A more widely used methodology for measuring force in single filaments is the indirect method, based on an internal load imposed on actin filaments by an anchored actin binding protein in a conventional *in vitro* motility assay. Warshaw et al. (1990) first demonstrated this principle using N-ethylmaleimide (NEM)-treated heavy meromyosin (HMM) and p-phenylenedimaleimide (pPDM)-treated HMM, while Janson et al. (1992) showed that a-actinin and filamin can also stop filament movement. The greater the myosin motor force on an actin filament, the higher the concentration of actin-binding protein needed to stop movement. Bing and Marston first demonstrated that the effect of a-actinin on the fraction of filaments moving is a consistent and sensitive method that can detect changes in isometric force of single actin filaments interacting with immobilized HMM (Bing et al., 2000). The initial studies investigated how tropomyosin and troponin may control force but only measured relative force, however, an analysis of how retardation works by Greenberg and Moore (2010) showed that it was possible to extract quantitative data from such measurements.

**FIGURE 5:**
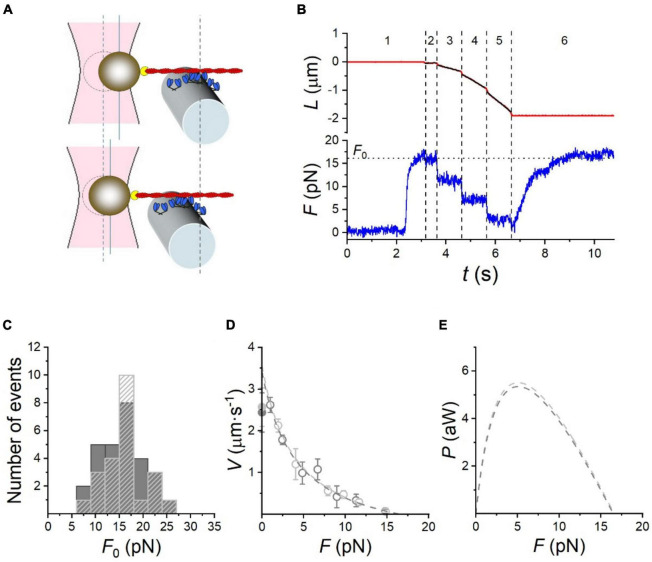
Performance of the rabbit HMM-based nanomachine. **(A)** Schematic representation of two snapshots during the interaction between the actin filament and the motor ensemble. Upper panel: in position clamp at F0; lower panel: in force clamp at 0.4 F0. **(B)** Recording of the actin filament sliding (L, upper trace, red) and force (F, lower trace, blue) during an interaction. Numbers bounded by dashed lines identify the different time intervals (it) as detailed in the text. **(C)** Frequency distribution of F0. Data are plotted in classes of 3 pN; dark gray bars, measurements in 0.1 mM CaCl2 ( = 77 M free [Ca^2 +^]); light gray dashed bars, in the absence of Ca^2 +^. **(D)** F-V relation in 0.1 mM CaCl_2_ (dark gray open circles) and in Ca^2 +^-free solution (light gray open circles). Points are mean ± SD from individual experiments, grouped in classes of force 0.15 F0 wide. Dark and light gray filled symbols on the ordinate are the Vf in the in vitro motility assay (IVMA) on rabbit HMM with and without Ca^2 +^, respectively. The dashed lines are Hill’s hyperbolic equation fits to the data with the same color code as symbols. Data in c and d are from 28 experiments in 0.1 mM CaCl_2_ and 23 experiments in Ca^2 +^-free solution. **(E)** Power (P) versus F, calculated from the corresponding F-V fits in **(D)**, with the same color code. Reproduced from Pertici et al. (2020) with permission.

The optical trap has been used for many years to measure very small movements and has been mainly applied to single molecule studies of unloaded contractility. To measure force needs a much stiffer optical trap which can be limiting (Veigel et al., 1998, 2003). Recently the optical trap method has been used for force measurements of the interaction of actin with a small ensemble of myosin molecules. There is a trade-off here between the maximum amount of force the trap can handle and the minimum number of myosin-actin interactions needed for continuous movement. Skeletal muscle has a low “duty ratio” which means that if the steady-state number of interactions is too low the filaments can detach from the actin at physiological ATP concentrations. Kaya et al. (2017) used whole myosin filaments, but in order to reduce the interactions to not overload the optical trap the filaments were copolymers of myosin and myosin rod (1:200 ratio) and the estimated steady-state number of interactions was just 37 in a 600 nm myosin filament. Pertici et al. (2020) coated HMM onto a microfiber at an appropriate dilution that enabled a minimum of 32 steady state interaction (Figure 5). Pertici et al. suggest the geometry of their setup is superior to that of Kaya et al. (2017) since the actin and HMM can be adjusted to exert force in a linear rather than oblique direction and because the HMM, being concentrated on a small surface, has a constant area of actin-myosin overlap unlike the two-filament system where the length of overlap is a variable. On the other hand, the two-filament system using myosin filaments does have the correct orientation of myosin heads whereas in the “nanomachine” HMM is randomly orientated toward actin.

Both these methods found that myosin acts in a coordinated way when interacting with a single actin filament; maximum isometric force and a force-velocity curve were obtained (see Figure 5).

In general, single filament systems offer a potential tool for investigating muscle contractile and regulatory protein mechanisms and the effects of mutations and post-translational modifications on force production that could match the equivalent unloaded studies using the *in vitro* motility assay. So far such studies have been limited to the thick filament (Rassier and Leite, 2020; Pertici et al., 2021) with only one study using single filaments to study thin filament regulation (Ishii et al., 2019).

## Conclusion

Over the last 20 years, techniques for measuring force in single myofibrils or myofilaments have increasingly been used for fundamental studies of striated muscle contractile mechanisms and their regulation by Ca^2+^, post translational modifications and disease-related mutations. The techniques are exacting, and throughput is low but the results are accurate and provide detailed information of contractility at the subcellular level that cannot be obtained by any other method.

The key advantages of subcellular contractility measurement are twofold. Firstly, the contractile apparatus is directly studied and secondly, rapid solution changing permits the measurement of the kinetics of the force changes induced by Ca^2+^ concentration changes for the first time.

These methods have potential for investigating regulation, post translational modifications and mutations in a huge variety of systems. The versatility of these systems plus the ability to combine force measurements with optical measurements allows the possibility of new types of experiments not yet envisaged. For instance, single myofibril and myofilament data can be valuable to form the basis of multiscale modeling of muscle (Månsson, 2019, 2020).

## Author Contributions

The author confirms being the sole contributor of this work and has approved it for publication.

## Conflict of Interest

The author declares that the research was conducted in the absence of any commercial or financial relationships that could be construed as a potential conflict of interest.

## Publisher’s Note

All claims expressed in this article are solely those of the authors and do not necessarily represent those of their affiliated organizations, or those of the publisher, the editors and the reviewers. Any product that may be evaluated in this article, or claim that may be made by its manufacturer, is not guaranteed or endorsed by the publisher.
